# Hippocampus segmentation in CT using deep learning: impact of MR versus CT-based training contours

**DOI:** 10.1117/1.JMI.7.6.064001

**Published:** 2020-11-11

**Authors:** Annika Hänsch, Jan Hendrik Moltz, Benjamin Geisler, Christiane Engel, Jan Klein, Angelo Genghi, Jan Schreier, Tomasz Morgas, Benjamin Haas

**Affiliations:** a Fraunhofer MEVIS, Bremen, Germany; b Varian Medical Systems Imaging Laboratory GmbH, Baden, Switzerland; c Varian Medical Systems Finland Oy, Helsinki, Finland; d Varian Medical Systems, Las Vegas, Nevada, United States

**Keywords:** radiotherapy planning, hippocampus, segmentation, deep learning, training data quality

## Abstract

**Purpose:** Hippocampus contouring for radiotherapy planning is performed on MR image data due to poor anatomical visibility on computed tomography (CT) data. Deep learning methods for direct CT hippocampus auto-segmentation exist, but use MR-based training contours. We investigate if these can be replaced by CT-based contours without loss in segmentation performance. This would remove the MR not only from inference but also from training.

**Approach:** The hippocampus was contoured by medical experts on MR and CT data of 45 patients. Convolutional neural networks (CNNs) for hippocampus segmentation on CT were trained on CT-based or propagated MR-based contours. In both cases, their predictions were evaluated against the MR-based contours considered as the ground truth. Performance was measured using several metrics, including Dice score, surface distances, and contour Dice score. Bayesian dropout was used to estimate model uncertainty.

**Results:** CNNs trained on propagated MR contours (median Dice 0.67) significantly outperform those trained on CT contours (0.59) and also experts contouring manually on CT (0.59). Differences between the latter two are not significant. Training on MR contours results in lower model uncertainty than training on CT contours. All contouring methods (manual or CNN) on CT perform significantly worse than a CNN segmenting the hippocampus directly on MR (median Dice 0.76). Additional data augmentation by rigid transformations improves the quantitative results but the difference remains significant.

**Conclusions:** CT-based training contours for CT hippocampus segmentation cannot replace propagated MR-based contours without significant loss in performance. However, if MR-based contours are used, the resulting segmentations outperform experts in contouring the hippocampus on CT.

## 1 Introduction

Radiotherapy (RT) is a medical discipline that requires segmentation of many anatomical structures for treatment planning. Based on the delineated targets and organs at risk (OAR), a treatment plan is calculated using a planning computed tomography (CT) scan of the patient. However, due to low soft-tissue contrast in CT, not all of the required structures are well visible on the scan. Therefore, structure segmentation based on magnetic resonance (MR) image data is often part of the RT workflow. One of these structures is the hippocampus, a compound structure in the brain. Eekers et al.^1^ describe MR-based contouring of the hippocampus as “essential” for RT planning.

From a clinical perspective, the hippocampus is an important OAR because of its role in short-term learning and memory. Radiation can negatively affect these functions leading to lower quality of life.^2^ These radiation-induced side effects can be reduced by hippocampal avoidance during whole brain RT.^3^^–^^5^ Therefore, for an automated treatment planning, accurate auto-segmentation of the hippocampus is highly desirable.

Segmentation of the hippocampus on MR data is an active research topic, probably also due to the link between the structure’s morphology and Alzheimer’s disease.^6^ Dill et al.^7^ reviewed existing methods and found that most of them rely on atlas-based techniques or deformable models. Since then, deep learning methods have been employed for the task in various variations.^8^^–^^11^

In the RT application, CT-only auto-contouring could simplify the clinical workflow by removing the need for an MR scan during treatment planning and for additional steps such as MR/CT registration. Zhao et al.^12^ proposed to perform whole brain segmentation including the hippocampus by generating synthetic MR from CT images with deep learning, then applying MR-based segmentation algorithms from literature. They reported mean Dice scores of 0.56/0.62 (left/right) on hippocampus segmentation. Recently, Porter et al.^13^ published a study of directly segmenting the hippocampus on non-contrast CT using a specific convolutional neural network (CNN). Their CNN, trained and evaluated on 390 patients, achieved a Dice score of 0.738/0.737 (left/right), only using CT data at inference time. However, both methods rely on the availability of paired MR and CT data during training of the CT-only (at inference time) methods. Zhao et al.^12^ use paired data to train the CT to MR converter. Porter et al.^13^ use training contours that were generated on MR and propagated to the corresponding CT using a registration.

The main question investigated in this study is whether or not we can also remove the need for MR data during training of a CNN that only uses CT data at inference time. In addition to the benefits of CT-only inference, this would also reduce the need for collecting paired MR/CT data which we have experienced to be difficult. In detail, we make the following contributions.

1. We compare the quality of hippocampus contours drawn on CT to ground truth contours drawn on MR data.
2. We compare the performance of CNNs for CT hippocampus segmentation trained on high quality propagated MR-based training contours with CNNs trained lower quality CT-based training contours.
3. We evaluate the model uncertainty of CNNs trained using the two training sets of different contour quality using Monte Carlo dropout as Bayesian approximation.^14^

As we can expect the manual contouring quality of the hippocampus on CT to be lower than on MR, one of the main questions is whether or not a CNN can make use of possibly inconsistent training segmentations and still produce consistent, high quality segmentation results. This is motivated by the success of the “student beats the teacher” approach, where automatically generated, and partially erroneous, segmentations were used to train a CNN which then outperformed the trainer algorithm on manually annotated ground truth.^15^

## 2 Methods

### 2.1 Data Preparation

#### 2.1.1 Database

A total of 45 pairs of CT and T1 MR image volumes of patients undergoing RT treatment of brain tumors were used in this study. All CT data were acquired on a single scanner model with pixel spacings 0.68 to 1.17 mm and slice thicknesses 1 to 3 mm. The MR data were acquired on five different scanner models from two manufacturers with pixel spacings 0.39 to 1.17 mm and slice thicknesses 1 to 2 mm.

The data were split randomly into five folds of 27 training cases, nine validation cases, and nine test cases each, so that each case was part of exactly one test set.

#### 2.1.2 Hippocampus contouring

The left and right hippocampi were contoured on all 45 image pairs, split between a radiologist (N=18) and a medical technical radiology assistant (N=27), so that each image was contoured by exactly one annotator. The radiologist has ∼7  years of clinical expertise in the field of neuroradiology (university center); and the medical technical radiology assistant has 20 years of general radiological experience, including 7 years working in the clinical imaging routine. Both annotators have agreed on important anatomical landmarks to be considered during segmentation, using independent sample images.

**Fig. 1:**
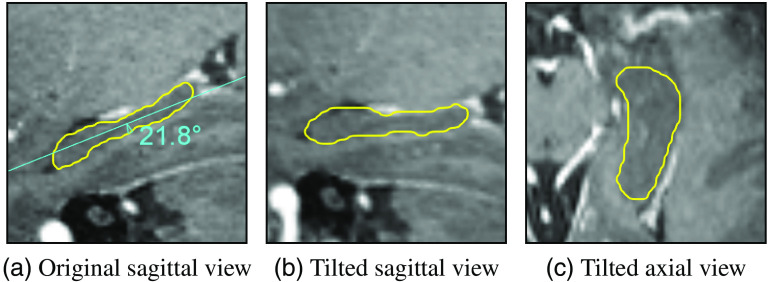
Hippocampus tilting for manual contouring. Yellow contours delineate the hippocampus on MR in (a) original and (b–c) tilted images.

First, all MR volumes were contoured, then all CT volumes were contoured with an interval of one week, to avoid use of anatomical information from the MR during contouring of the corresponding CT. This should simulate the absence of MR during contouring. For the manual contouring, all volumes were tilted (see Fig. 1) so that the hippocampus body was parallel to the tilted axial plane. Due to the course of the hippocampus being inclined by ∼20° in relation to the axial planes, the segmentation is significantly facilitated by doing so, as less slices need to be contoured. The tilt angles were determined manually for CT and MR. As the tilted images are only used for contouring but not for training (see Sec. 2.1.3), we do not expect the natural error in the manually determined tilt angle to impact the results of the training.

#### 2.1.3 Registration

Affine registration matrices for registering the CT and MR volume pairs were provided with the data. For each case, the quality of the available registration was verified and approved by the radiologist also involved in contouring. The verification was performed through blending the registered CT and MR images. As a result, six cases were excluded due to a missing or poor registration, resulting in the total of 45 cases used in this study.

The hippocampus contours drawn on tilted MR images were propagated to the corresponding CT images as follows: the contours were converted to binary masks and resampled to $1\times 1\times 1\;{\mathrm{mm}}^{3}$. The tilting was reverted and the affine registration was applied. The registered contours were postprocessed using Gaussian smoothing followed by thresholding to remove artifacts from the registration and resampling.

#### 2.1.4 Data preprocessing

The preprocessing for deep learning included resampling and intensity normalization. All data were resampled to 1.5-mm isotropic voxel size. The CT data were normalized to values in [0,1] within a window of Hounsfield units with center/width=40/80  HU. The MR data were normalized based on percentiles of image intensities where the second and 98th percentile were mapped to 0 and 1. All data preprocessing was performed in the software development framework MeVisLab.^16^

For basic data augmentation, random flipping of left and right body side was applied. All results reported in Secs. 3.2 and 3.3 use only this basic kind of data augmentation. To compensate for the limited data set size, selected trainings were repeated using additional data augmentation consisting of on-the-fly, normally-distributed rigid transformations: isotropic scaling with a standard deviation of 2.5%, and rotations in the axial, sagittal, and coronal plane with standard deviations of 5°, 5°, and 2.5°, respectively. The amount of augmentation was determined visually based on transformed images.

#### 2.1.5 Data postprocessing

Postprocessing of segmentations generated by deep learning was limited to binarization via thresholding at 0.5, selection of the largest connected component per side (left/right) and resampling back to ${1\times 1\times 1\;\mathrm{mm}}^{3}$ to compare against the reference contours.

#### 2.1.6 Overview of data sets

In total, we will use and refer to the following pairs of image data and contours throughout the rest of this study.

• MR/MR: MR image data with contours drawn on MR
• CT/CT: CT image data with contours drawn on CT
• CT/MRregCT: CT image data with contours drawn on MR and propagated to CT.

We will always use contours drawn on MR as ground truth for segmentation evaluation, i.e., MR/MR (when segmenting on MR) or CT/MRregCT (when segmenting on CT). In Sec. 3, we will further refer to CNNs trained on the CT/CT data as ${\mathrm{CT}/\mathrm{CT}}_{cnn}$ and to manually drawn contours as ${\mathrm{CT}/\mathrm{CT}}_{man}$ etc.

### 2.2 Deep Learning

#### 2.2.1 Convolutional neural network

We trained a custom 3D encoder-decoder CNN with one input channel (CT or MR) and three output channels (background, left, and right hippocampus). The starting architecture was the 2D One Hundred Layers Tiramisu^17^ architecture with four resolution levels, which makes use of dense convolutional blocks.^18^ It was extended to 3D by inserting an additional separable 3D convolution (3×3×1 followed by 1×1×3 in x×y×z) between 2D dense block and 2D max pooling. We always used padded 3×3×1 convolutions in xy dimensions and unpadded 1×1×3 convolutions in z dimension. This means that the CNN output size equals the input size in xy (256×256 voxels) but is smaller in z (7 slices versus 1 slice). This type of hybrid 2D/3D convolution approach has previously been used for head and neck segmentation.^19^ It allows to use 3D context while not requiring the computational power of a full 3D CNN such as the 3D U-Net^20^^,^^21^ or V-Net.^22^ The final layer was a softmax layer to generate class labels. Batch normalization^23^ and dropout^24^ were used for regularization. The use of dropout in the architecture is also relevant for model uncertainty estimation (see Sec. 2.3.2). The architecture was implemented in keras.^25^

#### 2.2.2 Training parameters

All CNNs were trained using a hyperparameter search to overcome training instability issues and to reduce random model noise from the stochastic nature of the model initialization and training. The random weight initialization and learning rate were the only optimized hyperparameters and were randomly sampled, from a logarithmic scale in case of the learning rate. The hypersearch was implemented using the HpBandSter python package^26^ with the HyperBand option.^27^ In each stage of the search, we trained the following number of CNNs (with number of total training iterations in brackets): 16 (5 k), 8 (10 k), 4 (20 k), 2 (40 k), and 1 (80 k). The best CNN of each search was chosen based on the highest Jaccard coefficient during the validation step. The Adam optimizer^28^ was used for optimization of the Dice loss function.^22^

For each choice of image/contour combination (see Sec. 2.1.6), the hyperparameter search was repeated five times using the five different splits into training, validation and test data as described in Sec. 2.1.1. The test data of the respective fold were only used for evaluation once the final best model of each search had been chosen based on the validation set. For simplicity, in Sec. 3, we will refer to the set of five CNNs trained per image/contour data set simply as CNN on the respective data set. A split into five folds was chosen as a trade-off between training data set size and required training time, which is increased due to the use of the hyperparameter search described above.

### 2.3 Evaluation

#### 2.3.1 Segmentation performance

We used the following well-known and common metrics to evaluate the similarity of two segmentations (manually or automatically generated): Dice score, mean surface distance, and Hausdorff distance. In addition, the contour Dice score^29^ was used which measures the fraction of the axial contours that lie within a predefined tolerance (here 1, 3, 5, 7, and 10 mm) of the reference contour. The metric is a contour-based version of the surface Dice score,^19^ as corrections in RT planning are typically based on contours, not on surfaces. In analogy to Porter et al.,^13^ we also computed the passing rate of the Radiation Therapy Oncology Group (RTOG) 0933 trial, which is defined as the percentage of cases with Hausdorff distance ≤7  mm. All metrics were computed on the 3D volumes and separately for left and right hippocampus. For comparing the performance of different contour creation methods (manual or CNN) and data augmentation strategies, we used the Wilcoxon signed rank test implemented in SciPy.^30^

#### 2.3.2 Uncertainty estimation

To estimate model uncertainty, Bayesian dropout^14^ was used with N=25 samples during inference to calculate an entropy map U. A high entropy value suggests that the CNN is uncertain about the classification of the given voxel. For segmentation, the uncertainty is typically elevated close to the contour of the predicted segmentation. Therefore, we computed the uncertainty density within a tolerance τ around the predicted contour c in the discrete image voxel grid I as

$$U_{\tau }=\underset{x\in D_{\tau }}{\sum }U(x)\cdot {\left|D_{\tau }\right|}^{-1}\;\text{with}\;D_{\tau }=\{x\in I|{\left\|x-c\right\|}_{2}\leq \tau \}.$$ (1)

Tolerances of k·1.5  mm with k=1,…,5 correspond to multiples of the CNN’s operating resolution at 1.5-mm isotropic voxel size.

## 3 Results

This section is made up of four parts : first, we compare the manual contours drawn on MR with those drawn on CT. Then, we compare the performance of CNNs trained using these different contours. For reference, we also compare to a CNN segmenting the hippocampus on MR. We then evaluate the model uncertainty of selected CNNs. Finally, we re-evaluate the CT segmentation trained using additional data augmentation.

### 3.1 Comparison of Manual CT and MR Contours

**Fig. 2:**
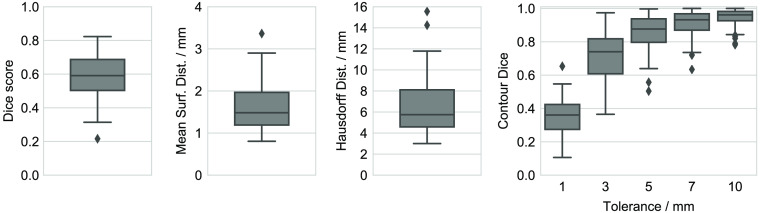
Quantitative comparison of manual CT and MR training contours. Metrics shown are (from left to right) Dice score, mean surface distance, Hausdorff distance, and contour Dice score for multiple tolerances.

Figure 2 shows various metrics comparing the manual MR and CT contours, where the MR-based contour is considered as ground truth in this study. In 62% of cases, the Hausdorff distance is below the 7-mm acceptance threshold. The contour Dice score, with a median value 0.93 for 7-mm tolerance, shows that this threshold is typically exceeded only for a small fraction of the contour. However overall, with a median Dice score of 0.59 with interquartile range (IQR) 0.19 (mean 0.58±0.13) and a median Hausdorff distance of 5.75 mm with IQR 3.52 mm (mean 6.50±2.59  mm), the manual CT contours deviate considerably from the MR contours. For comparison, Liedlgruber et al.^31^ report mean inter-observer Dice scores of 0.76±0.07 and mean Hausdorff distances of 6.50±1.30  mm in a three-observer study on MR hippocampus segmentation. This supports our underlying assumption that CT-based contours are subject to higher uncertainty than MR-based contours.

**Fig. 3:**
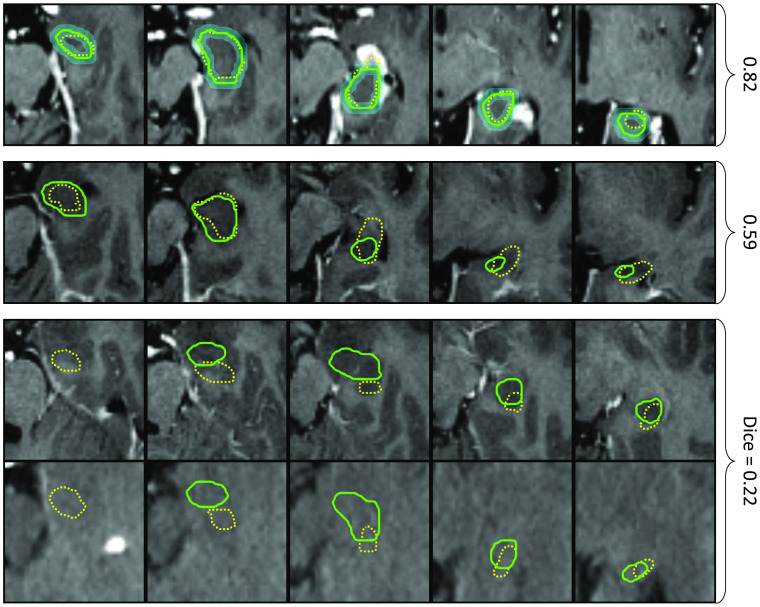
Exemplary cases with the highest (top row), median (middle row) and lowest (bottom rows) Dice scores between manual MR (green solid) and manual CT (yellow dotted) contours. The contours are shown on MR and for the last case also on CT, both in their original coordinate system. The cyan overlay in the first row indicates a tolerance region of 2 mm around the MR contour. The latter is considered as ground truth in this study.

Figure 3 shows examples of manual CT and MR segmentations with high, median, and low Dice score. It is noteworthy that the soft tissue contrast is much lower in CT image data, which according to the contouring radiologist, results in requiring a mental model of the hippocampus while delineating it on CT.

The main question of this study is if, despite lower training contour quality when generating reference contours on CT, we can train CNNs on ${\mathrm{CT}/\mathrm{CT}}_{man}$ and ${\mathrm{CT}/\mathrm{MRregCT}}_{man}$ contours with similar performance.

### 3.2 Training on Manual CT or Manual MR Contours

In this section, we compare segmentation results of the ${\mathrm{CT}/\mathrm{CT}}_{cnn}$ versus the ${\mathrm{CT}/\mathrm{MRregCT}}_{cnn}$, each evaluated against the ${\mathrm{CT}/\mathrm{MRregCT}}_{man}$ ground truth labels. For the results presented in this section, only basic data augmentation (flipping of left and right body side) was used. For reference, we also include segmentation results of the ${\mathrm{MR}/\mathrm{MR}}_{cnn}$ and the ${\mathrm{CT}/\mathrm{CT}}_{man}$ segmentations already presented in Sec. 3.1. The ${\mathrm{MR}/\mathrm{MR}}_{cnn}$ should serve as an estimation of an upper limit to the segmentation performance with the available data. Given the much better visibility of the hippocampus on MR than CT image data, we do not expect the segmentation performance to be higher on CT than on MR. Each of the five models from each five-fold cross-validation is evaluated on its respective test set of nine test cases (which is disjoint from the other test sets), see Sec. 2.2.2. In the following, we will always jointly report the test results on all 45 test cases from the five models per cross-validation.

**Fig. 4:**
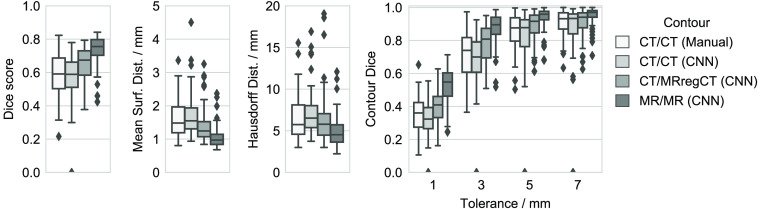
Comparison of different hippocampus contours (manually and automatically generated) against the ground truth using Dice score, mean surface distance, Hausdorff distance, and contour Dice score. For the surface distances, three outliers with Dice score 0 for ${\mathrm{CT}/\mathrm{CT}}_{cnn}$ are not shown.

**Fig. 5:**
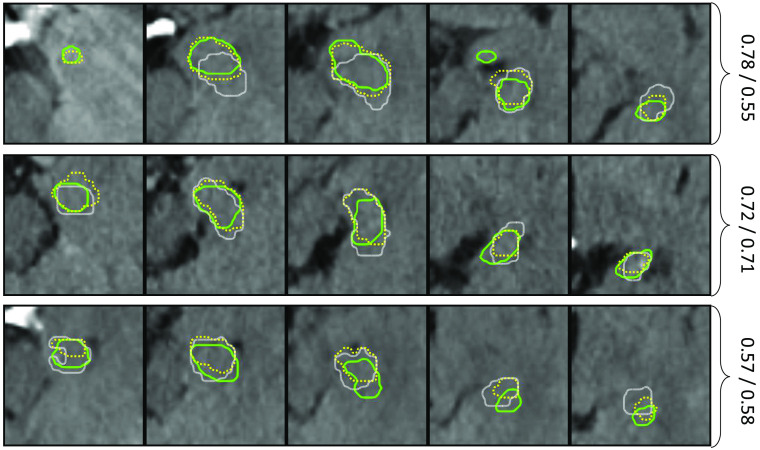
Segmentation results of ${\mathrm{CT}/\mathrm{MRregCT}}_{cnn}$ (yellow dotted) and ${\mathrm{CT}/\mathrm{CT}}_{cnn}$ (white finely dashed) against the ground truth (green solid). The numbers on the right indicate the Dice scores of training on MR-based (and propagated to CT) versus CT-based contours. The rows correspond to cases with high, median and lower 25% Dice score for training on MR contours. The middle and bottom row also correspond to top 25% and median Dice scores for training on CT contours.

Figure 4 shows performance metrics comparing each of the contours against the ground truth labels drawn on MR, where the comparison of ${\mathrm{CT}/\mathrm{MRregCT}}_{cnn}$ versus ${\mathrm{CT}/\mathrm{CT}}_{cnn}$ is our main focus of interest. The former significantly outperforms the latter with respect to the Dice score (median value 0.674 versus 0.589, $p=4.8\cdot 10^{-12}$). The median surface distance metrics are lower when training on MR versus CT contours (mean surface distance 1.3 mm versus 1.5 mm, Hausdorff distance 5.8 mm versus 6.5 mm). This also results in a much higher RTOG passing rate (cases with Hausdorff distance below 7 mm^13^) of 74.4% versus only 54.4%. It is noteworthy that in three cases, ${\mathrm{CT}/\mathrm{CT}}_{cnn}$ results in a zero Dice score due to a large false positive being selected by the largest connected component postprocessing. The differences in contour Dice scores are large especially for small and medium tolerances. This means that while both CNNs can detect the approximate location of the hippocampus within the brain, ${\mathrm{CT}/\mathrm{MRregCT}}_{cnn}$ can segment the hippocampus with higher accuracy. For illustration, Fig. 5 shows exemplary cases at different performance levels with contours of both CNNs versus the ground truth. In summary, the results show that training on the lower quality manual CT contours does not reach the performance of training on the contours generated on MR data.

Compared to the $CT/CT_{\text{man}}$ contouring (“teacher”), the $CT/CT_{\text{cnn}}$ (“student”) performance is comparable (median Dice score 0.589 versus 0.591, p=0.84). Based on surface distances and the contour Dice, the manual contours slightly outperform the CNN, however except for Hausdorff distance and contour Dice with tolerances 1 and 10 mm, these differences are not significant. This means that the student beats the teacher^15^ principle does not hold in this case.

Compared to the ${\mathrm{MR}/\mathrm{MR}}_{cnn}$ which serves as an upper performance estimate as described above, the ${\mathrm{CT}/\mathrm{MRregCT}}_{cnn}$ still performs significantly worse with respect to all metrics (median Dice score 0.756 versus 0.674, $p=6.4\cdot 10^{-13}$). The RTOG passing rates are 87.8% and 74.4%, respectively. The observed ${\mathrm{MR}/\mathrm{MR}}_{cnn}$ performance is similar to the inter-observer variability for MR hippocampus segmentation reported in literature.^31^

### 3.3 Uncertainty Evaluation

**Fig. 6:**
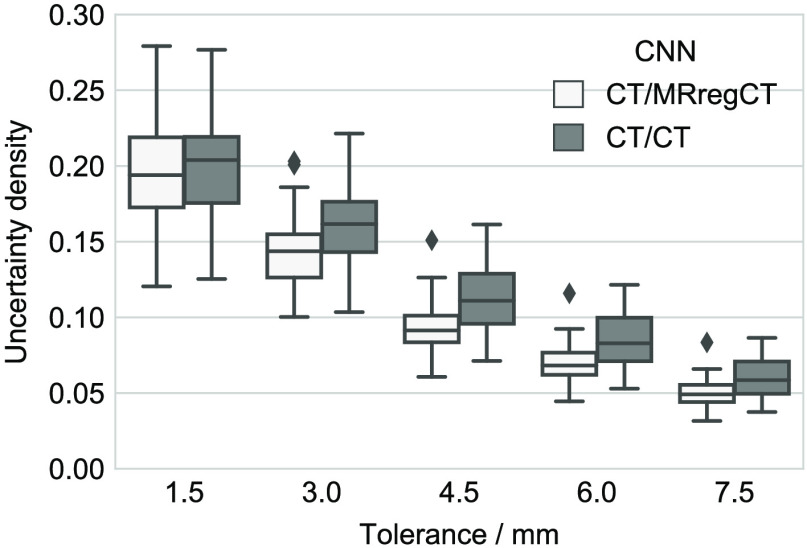
Uncertainty density within a tolerance region around the predicted contours (only true positive connected components of the segmentation mask) by ${\mathrm{CT}/\mathrm{MRregCT}}_{cnn}$ and ${\mathrm{CT}/\mathrm{CT}}_{cnn}$.

**Fig. 7:**
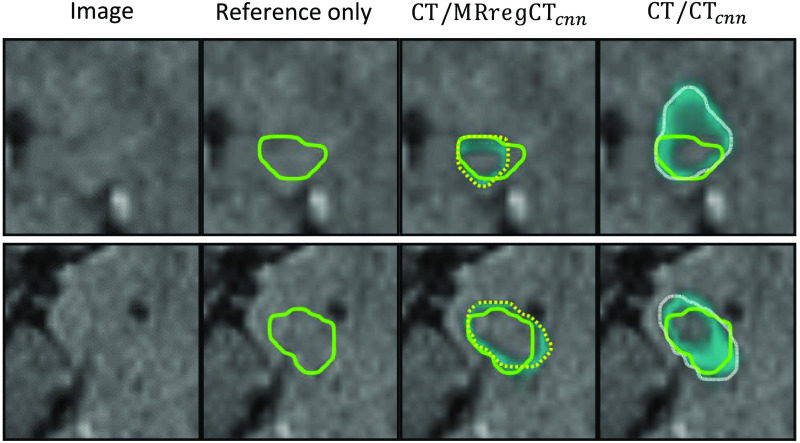
Visualization of the uncertainty map (cyan overlay) for ${\mathrm{CT}/\mathrm{MRregCT}}_{cnn}$ (yellow dashed) and ${\mathrm{CT}/\mathrm{CT}}_{cnn}$ (white dotted) compared to the ground truth (green solid) for two different cases. Uncertainty is often high close to the contour of the predicted segmentation.

Figure 6 shows the uncertainty density of ${\mathrm{CT}/\mathrm{MRregCT}}_{cnn}$ and ${\mathrm{CT}/\mathrm{CT}}_{cnn}$ within a tolerance around their predicted contours as described in Sec. 2.3.2. One can make multiple observations: First of all, as expected, the uncertainty density is highest close to the contour. Since this uncertainty about the precise contour location often also applies to reference segmentations considered as ground truth, such small deviations are typically not clinically relevant. Second, the uncertainty of the ${\mathrm{CT}/\mathrm{CT}}_{cnn}$ is significantly higher than of the ${\mathrm{CT}/\mathrm{MRregCT}}_{cnn}$ for all tolerances τ≥3  mm. This means that training on CT contours not only reduces the performance but also increases the uncertainty of the resulting CNN compared to training on contours drawn on MR. Figure 7 shows examples of model uncertainty which extends past the close vicinity of the predicted contour. In some but not all cases do falsely segmented regions coincide with areas of high uncertainty. Figure 7 also once again illustrates the difficulty of the task, as the hippocampus is hardly visible in the given CT contrast.

### 3.4 Training with Additional Data Augmentation

**Fig. 8:**
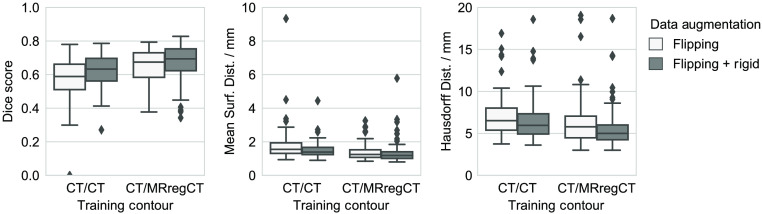
Segmentation performance depending on training contour set and data augmentation method. Some outliers are cut off for better visibility.

Figure 8 shows results of the ${\mathrm{CT}/\mathrm{CT}}_{cnn}$ and ${\mathrm{CT}/\mathrm{MRregCT}}_{cnn}$ cross-validation re-trained with additional rigid data augmentation as described in Sec. 2.1.4. For both CT-based and MR-based training contours, additional data augmentation improves the segmentation performance. All pair-wise differences in the median Dice score of the four training configurations are significant. The same holds for both surface distance measures except for the Hausdorff distance of ${\mathrm{CT}/\mathrm{CT}}_{cnn}$ (additional data augmentation) versus ${\mathrm{CT}/\mathrm{MRregCT}}_{cnn}$ (flipping only). Moreover, the median Dice score of the ${\mathrm{CT}/\mathrm{CT}}_{cnn}$ with additional data augmentation is significantly higher than of the manual CT contours (0.633 versus 0.591, p=0.048). However, in this comparison, differences in the surface distance measures are not significant. Table 1 summarizes the median per-case improvement for both training contour configurations that is achieved using more data augmentation. For CT-based training contours, the benefit of using additional data augmentation is slightly higher than for MR-based contours, shown by a higher median improvement in all metrics.

**Table 1:** Median (and IQR) of the per-case improvement when training with additional data augmentation compared to basic flipping augmentation.

Training contour	Dice score	Mean surface distance (mm)	Hausdorff distance (mm)
CT/CT	+0.039 (0.058)	−0.143 (0.257)	−0.566 (2.218)
CT/MRregCT	+0.023 (0.047)	−0.083 (0.204)	−0.307 (2.019)

## 4 Discussion

Our results suggest that deep learning-based CT hippocampus segmentation trained on propagated MR contours outperforms training based on CT contours and also experts contouring directly on CT. There is a bias in these results, as the contours that were used as ground truth are also propagated from the MR. It can be expected that, if training and test set contours are from the same source (here: from the same mode of contouring), the match between predicted segmentations and ground truth will be higher than if the two data sets are from different sources. However, results from the uncertainty evaluation also suggest that the CNN trained on CT contours is more uncertain than the one trained on MR contours. We suppose that the higher inconsistency and overall lower quality of the CT contours results in a higher model uncertainty and lower segmentation accuracy.

Based on the results in Sec. 3.2, the student beats the teacher approach^15^ discussed in Sec. 1 does not hold in this scenario, as the expert contouring on CT (the teacher) and the CNN learning on these contours (the student) are on par compared to the MR-based ground truth. One reason might be the relatively small training set size with only 27 cases actually used for training in each fold. Ghafoorian et al.^15^ used 246 cases for training, so ∼10 times as many. It could be that more training examples are required to account for the inconsistency in the training data, assuming that any errors are not systematic. The results in Sec. 3.4 show that training with additional (rigid) data augmentation can leads to a small but significant improvement of the ${\mathrm{CT}/\mathrm{CT}}_{cnn}$ compared to the manual CT-based contours in the Dice score but not in surface distance measures. However, also with more data augmentation, the segmentation performance using CT-based contours does not reach that of using MR-based contours for training. Then again, the improvement due to data augmentation is larger for CT-based than MR-based training contours. Future work should therefore investigate if a larger training set and more advanced data augmentation can close this gap in performance.

The poor visibility of the hippocampus on CT raises the question how good the segmentation directly on CT can be compared to segmenting it on MR. We suppose that the CT CNNs in our study mainly need to rely on features of neighboring structures, such as lateral ventricles, brain stem, and basal cisterns. An accurate segmentation will therefore probably be possible only within a fairly large tolerance region. In this study, we observed a large performance margin (median Dice score 0.756 versus 0.694) for segmenting the hippocampus on MR versus CT image data (using the best CNN each). However, this may also include a bias, as the MR data was acquired on multiple scanners, while the CT data were from a single scanner model. Porter et al.^13^ report a median Dice score of 0.74 after training on a significantly larger and high resolution CT data set (390 patients in total for training and test with MR-based annotations), which is similar to the performance that we reached on MR image data.

Furthermore, what we have neither investigated nor discussed so far is the use of techniques such as transfer or representation learning. Segmentation performance on CT might improve, if a CNN was pre-trained to learn an (ideally modality independent) feature representation of the hippocampus which could be transferred to the CT-only training. This would most likely also require CT and MR data during training (possibly unpaired), but could further improve segmentation performance.

Overall, the focus of this study was on the training data, differences in its quality depending on the modality used for annotation and consequences for CNN training. Therefore we did not discuss or evaluate the chosen architecture in detail, for example, compared to other 2D, 2D/3D, or 3D approaches from literature. For future work, it would be interesting to reproduce our results on a commonly used architecture such as the 2D or 3D U-Net.^20^^,^^22^ The 2D versus 3D comparison could yield further insights with respect to hippocampus segmentation on CT versus MR data in general. For CT segmentation, where the hippocampus visibility is lower than on MR data, the use of 3D context might be more relevant.

## 5 Conclusion

CNNs are capable of segmenting the hippocampus directly on CT with good accuracy, but our results show that this requires high quality and anatomically accurate training contours generated on MR and propagated to CT. Replacing such MR-based contours by CT-based ones significantly reduces the segmentation performance of the trained CNNs and also increases their Bayesian dropout uncertainty. Despite the relatively small training data set size, the CNN trained on high quality MR-based training contours outperforms medical experts in contouring the hippocampus directly on CT. Using a larger training set in addition to more data augmentation might further improve the segmentation performance.

## References

[1] Eekers D. B. P. et al., “The EPTN consensus-based atlas for CT- and MR-based contouring in neuro-oncology,” Radiother. Oncol. 128(1), 37–43 (2018). RAONDT 0167-8140 10.1016/j.radonc.2017.12.013 29548560

[2] Monje M. L. Palmer T., “Radiation injury and neurogenesis,” Curr. Opin. Neurol. 16(2), 129–134 (2003). 10.1097/00019052-200304000-00002 12644738

[3] Gondi V. Tomé W. A. Mehta M. P., “Why avoid the hippocampus? A comprehensive review,” Radiother. Oncol. 97(3), 370–376 (2010). RAONDT 0167-8140 10.1016/j.radonc.2010.09.013 20970214 PMC2997490

[r4] Gondi V. et al., “Preservation of memory with conformal avoidance of the hippocampal neural stem-cell compartment during whole-brain radiotherapy for brain metastases (RTOG 0933): a phase II multi-institutional trial,” J. Clin. Oncol. 32(34), 3810–3816 (2014). JCONDN 0732-183X 10.1200/JCO.2014.57.2909 25349290 PMC4239303

[5] Gondi V. et al., “Preservation of neurocognitive function (NCF) with conformal avoidance of the hippocampus during whole-brain radiotherapy (HA-WBRT) for brain metastases: preliminary results of phase III trial NRG oncology CC001,” Int. J. Radiat. Oncol. Biol. Phys. 102(5), 1607 (2018). IOBPD3 0360-3016 10.1016/j.ijrobp.2018.08.056

[6] Jack C. R. et al., “MR-based hippocampal volumetry in the diagnosis of Alzheimer’s disease,” Neurology 42(1), 183–188 (1992). NEURAI 0028-3878 10.1212/WNL.42.1.183 1734300

[7] Dill V. Franco A. R. Pinho M. S., “Automated methods for hippocampus segmentation: the evolution and a review of the state of the art,” Neuroinformatics 13(2), 133–150 (2015).1539-2791 10.1007/s12021-014-9243-4 26022748

[8] Chen Y. et al., “Accurate and consistent hippocampus segmentation through convolutional LSTM and view ensemble,” Lect. Notes Comput. Sci. 10541, 88–96 (2017). LNCSD9 0302-9743 10.1007/978-3-319-67389-9_11

[r9] Chen Y. et al., “Hippocampus segmentation through multi-view ensemble ConvNets,” in IEEE 14th Int. Symp. Biomed. Imaging (ISBI 2017), pp. 192–196 (2017). 10.1109/ISBI.2017.7950499

[r10] Thyreau B. et al., “Segmentation of the hippocampus by transferring algorithmic knowledge for large cohort processing,” Med. Image Anal. 43, 214–228 (2018). 10.1016/j.media.2017.11.004 29156419

[11] Ataloglou D. et al., “Fast and precise hippocampus segmentation through deep convolutional neural network ensembles and transfer learning,” Neuroinformatics 17(4), 563–582 (2019).1539-2791 10.1007/s12021-019-09417-y 30877605

[12] Zhao C. et al., “Whole brain segmentation and labeling from CT using synthetic MR images,” Lect. Notes Comput. Sci. 10541, 291–298 (2017). LNCSD9 0302-9743 10.1007/978-3-319-67389-9_34

[13] Porter E. et al., “Hippocampus segmentation on noncontrast CT using deep learning,” Med. Phys. 47(7), 2950–2961 (2020). MPHYA6 0094-2405 10.1002/mp.14098 32065401

[14] Gal Y. Ghahramani Z., “Dropout as a Bayesian approximation: representing model uncertainty in deep learning,” in ICML’16: Proc. 33rd Int. Conf. Mach. Learn., Vol. 48, pp. 1050–1059 (2016).

[15] Ghafoorian M. et al., “Student beats the teacher: deep neural networks for lateral ventricles segmentation in brain MR,” Proc. SPIE 10574, 105742U (2018). PSISDG 0277-786X 10.1117/12.2293569

[16] Ritter F. et al., “Medical image analysis: a visual approach,” IEEE Pulse 2(6), 60–70 (2011). 10.1109/MPUL.2011.942929 22147070

[17] Jégou S. et al., “The one hundred layers tiramisu: fully convolutional DenseNets for semantic segmentation,” in IEEE Conf. Comput. Vision and Pattern Recognit. Workshops, pp. 1175–1183 (2017).

[18] Huang G. et al., “Densely connected convolutional networks,” in IEEE Conf. Comput. Vision and Pattern Recognit., pp. 2261–2269 (2017). 10.1109/CVPR.2017.243

[19] Nikolov S. et al., “Deep learning to achieve clinically applicable segmentation of head and neck anatomy for radiotherapy,” Technical Report, arXiv:1809.04430 (2018).

[20] Ronneberger O. Fischer P. Brox T., “U-Net: convolutional networks for biomedical image segmentation,” Lect. Notes Comput. Sci. 9351, 234–241 (2015). LNCSD9 0302-9743 10.1007/978-3-319-24574-4_28

[21] Çiçek Ö. et al., “3D U-Net: learning dense volumetric segmentation from sparse annotation,” Lect. Notes Comput. Sci. 9901, 424–432 (2016). LNCSD9 0302-9743 10.1007/978-3-319-46723-8_49

[22] Milletari F. Navab N. Ahmadi S.-A., “V-Net: fully convolutional neural networks for volumetric medical image segmentation,” in IEEE Fourth Int. Conf. 3D Vision, pp. 565–571 (2016). 10.1109/3DV.2016.79

[23] Ioffe S. Szegedy C., “Batch normalization: accelerating deep network training by reducing internal covariate shift,” in Proc. 32nd Int. Conf. Mach. Learn. Res., Vol. 37, pp. 448–456 (2015).

[24] Srivastava N. et al., “Dropout: a simple way to prevent neural networks from overfitting,” J. Mach. Learn. Res. 15(56), 1929–1958 (2014).

[25] Chollet F. et al., “Keras,” 2015, https://keras.io.

[26] Falkner S. Klein A. Hutter F., “BOHB: robust and efficient hyperparameter optimization at scale,” in Proc. 35th Int. Conf. Machine Learn., Vol. 80, pp. 1437–1446 (2018).

[27] Li L. et al., “Hyperband: a novel bandit-based approach to hyperparameter optimization,” J. Mach. Learn. Res. 18(185), 1–52 (2018).

[28] Kingma D. P. Ba J., “Adam: a method for stochastic optimization,” in 3rd Int. Conf. Learn. Represent., (2015).

[29] Moltz J. H. et al., “Learning a loss function for segmentation: a feasibility study,” in IEEE 17th Int. Symp. Biomed. Imaging, pp. 957–960 (2020). 10.1109/ISBI45749.2020.9098557

[30] Virtanen P. et al., “SciPy 1.0: fundamental algorithms for scientific computing in Python,” Nat. Methods 17(3), 261–272 (2020).1548-7091 10.1038/s41592-019-0686-2 32015543 PMC7056644

[31] Liedlgruber M. et al., “Variability issues in automated hippocampal segmentation: a study on out-of-the-box software and multi-rater ground truth,” in IEEE 29th Int. Symp. Comput.-Based Med. Syst., pp. 191–196 (2016). 10.1109/CBMS.2016.55

